# The Impact of Diplopia on Reading

**DOI:** 10.22599/bioj.122

**Published:** 2019-01-21

**Authors:** Beckie Lijka, Sonia Toor, Gemma Arblaster

**Affiliations:** 1 Academic Unit of Ophthalmology and Orthoptics, University of Sheffield, GB; 2 Sheffield Teaching Hospitals NHS Foundation Trust, GB

**Keywords:** diplopia, reading, Radner Reading Chart

## Abstract

**Aim:**

To compare the effect of induced vertical diplopia (small and large separation) on reading speed and accuracy.

**Methods:**

The Radner Reading Chart (RRC) was used to measure reading speed (correct words per minute (wpm)) and accuracy (percentage). Accuracy was measured using two different methods: ‘accuracy-omission’ where only the omission of a word reduced the score, and ‘accuracy-addition and omission’ where any error reduced the score. Three viewing conditions were created using Fresnel prisms on plano glasses: a control condition without diplopia (6 prism dioptres (Δ) base up (BU) over each eye), small separation vertical diplopia (3Δ BU right eye and 3Δ base down (BD) left eye) and large separation vertical diplopia (6Δ BU right eye and 6Δ BD left eye). Viewing conditions were counterbalanced to minimise order effects.

**Results:**

Twenty-four participants were included with a mean age of 20.1 years. The mean reading speed in the control condition was 156.90 wpm. Both diplopic conditions significantly reduced the reading speed compared to the control condition, small separation diplopia to 62.75 wpm (p < 0.001) and large separation diplopia to 105.71 wpm (p < 0.001). The mean reading speed with small separation diplopia was significantly slower than the mean reading speed with large separation diplopia (p < 0.01). Median accuracy scores in the control and the large separation diplopia conditions were 100% using both methods of measuring accuracy. The small separation diplopia condition significantly reduced accuracy to 92.86% (accuracy-omission method) and to 57.50% (accuracy-addition and omission method) compared to the control condition (p < 0.01) and the large separation diplopia condition (p < 0.05).

**Conclusion:**

When vertical diplopia was induced using Fresnel prisms, diplopia of smaller separation resulted in the greatest reduction in reading speed and accuracy, compared to without diplopia and large separation diplopia.

## Introduction

The impact of strabismus on quality of life (QOL) typically causes a patient to have emotional and psychological problems, yet symptoms of diplopia additionally lead to patient reports of and concerns about functional difficulties (Hatt et al. 2007; Hatt et al. 2009a; Hatt et al. 2009b; Leske, Hatt & Holmes 2010). In an attempt to objectively quantify diplopia symptoms Sullivan et al. (1992) scored diplopia using the Goldmann Perimeter. The highest weighting was given to diplopia in primary position and the reading position based on the assumption that diplopia in these positions had a greater impact on function. Holmes, Leske & Kupersmith (2005) used the cervical range of motion diplopia examination, which also gave a greater weighting to diplopia in primary position and the reading position. Holmes et al. (2013) devised a Diplopia Questionnaire for patients to self-report the frequency and position of diplopia, with a scoring method that also gave a higher weighting to diplopia in primary position and the reading position. Whilst the position of diplopia is a factor affecting functional ability, the impact of the amount of separation of diplopic images on functional ability is less well understood. Worsening diplopia can be used to describe an increase in diplopia separation (Spector 1990; Pelak 2004; Mehta, Iqbal & Bowman 2010). However, prism therapy to further separate diplopic images, if diplopia cannot be joined (Shainberg, Roper-Hall & Chung 1995; Rosenbaum & Santiago 1999) conversely suggests that a larger diplopia separation may have less of an impact on function and is preferable for some patients.

A common method of assessing the impact of visual conditions is to measure reading. Reading is a functional daily activity for most seeing adults and is a popular leisure activity participated in by over two thirds of the UK adult population (Randall 2013). Reading is commonly featured in health related QOL questionnaires, it makes up one of two visual function domains in the AS-20 (Leske et al. 2012) and is the second largest topic, after driving, addressed in the VF-14 (Sabri et al. 2006). When a condition impacts upon reading it reduces QOL (Uusitalo et al. 1999; Bavishi, Slade & Levy 2016) and specifically, reduction in reading speed has been correlated with reduced QOL (Hazel et al. 2000).

The Radner Reading Chart (RRC) was designed to provide a reliable and repeatable assessment of reading. It has been used in studies of nystagmus, low vision, amblyopia, macular conditions and cataract (Stifter et al. 2005a; Stifter et al. 2005b; Kiss et al. 2006; Finger et al. 2009; Burggraaff et al. 2010; Barot et al. 2013). The RRC contains three charts, each with 15 sentences, which reduce in print size in 0.1 logMAR intervals. The difficulty of the text is equal to third grade level (UK year 4) (Altpeter et al. 2015). Sentences are comparable in the number of words (14), lexical difficulty, syntactical complexity, word length, position of words and number of syllables. There is some repetition of sentences within and between the charts, but the same sentence is never repeated for the same text size (Radner & Diendorfer 2014). The RRC can measure four different parameters of reading performance: reading acuity, critical print size, maximum reading speed and mean reading speed. Mean reading speed, measured in words per minute (wpm) has been defined as a measurement of reading function (Hahn et al. 2006). Reading accuracy can be measured in different ways (Jeanes et al. 1997; Birch et al. 2010; Vurro, Crowell & Pezaris 2014) and has also been suggested to be an important measurement in stroke, strabismus and convergence insufficiency (Dusek, Pierscionek & Mcclelland 2011; Beasley & Davies 2013; Ridha, Sarac & Erzurum 2014). Although the RRC can take reading errors into account when calculating a reading acuity LogRAD-Score, it is not solely a measure of reading accuracy.

This study aimed to compare the effect of induced diplopia (small and large separation) on reading speed and accuracy using the RRC.

## Methods

### Participants

Ethical approval for the study was granted from the University of Sheffield Research Ethics Committee. Twenty-four student volunteers were recruited to take part in the study. The volunteers were a mixture of orthoptic and non-orthoptic undergraduate students. The inclusion criteria were: age range 18–35 years (to exclude presbyopic individuals); near and distance visual acuity (VA) of 0.1 logMAR or better in each eye (unaided or with contact lens correction to exclude amblyopia and allow participants to wear plano lenses); no vertical deviation or decompensating horizontal phoria detected on a cover test; and TNO stereoacuity 60” of arc or better (to exclude microtropia). Participants had to have no known condition that could impact reading ability.

### Viewing Conditions

Vertical diplopia was induced by using plano glasses fitted with vertical Fresnel prisms. Plano glasses were identical in style and size. The conditions created were control condition, small separation diplopia and large separation diplopia. In the control condition, Fresnel prisms were fitted 6 prism dioptres (Δ) base up (BU) to both lenses, so the participants still had binocular single vision (BSV). In the small separation diplopia condition, 3Δ BU and 3Δ base down (BD) Fresnel prisms were fitted to the right lens and left lens respectively. In the large separation diplopia condition 6Δ BU and 6Δ BD Fresnel prisms were fitted to the right lens and left lens respectively.

### Procedure

Following informed consent from each participant, written instructions were given explaining the task. For each viewing condition, participants were asked to read the 0.4 logMAR sentence at 40 cm. Instructions were to read the sentence ‘as quickly and accurately as possible without making any mistakes’ as soon as the sentence was uncovered and the experimenter said ‘go’. If a participant read an incorrect sentence, the experimenter waited until they finished and then read standardised instructions stating they had read the incorrect sentence and restating which sentence to read. Participants were instructed to keep both eyes open and their head still during the task. The task was audio recorded.

Participants completed the task under each of the three viewing conditions, using a different RRC each time. To minimise any order effects, the order of RRC presentation and viewing condition were counterbalanced so that each participant completed a different combination of viewing condition and chart order.

### Analysis

The experimenter was blind to the viewing condition when analysing the data from the audio recordings. Reading speed was measured in wpm using the method stated in the RRC: *Number of correct words read/time* (*seconds*) × *60*. The number of correct words was the number of words the participant said correctly and in the correct order from the 0.4 logMAR sentence (maximum 14). Words read in an incorrect order were not counted as correct. Time was calculated from the point the experimenter said ‘go’ to the last word said by the participant, this word did not need to be correct.

Two methods of measuring accuracy as a percentage were used, accuracy-omission and accuracy-addition and omission. Accuracy-omission (%) was calculated as: *Number of correct words read/number of words in the sentence (14) × 100*. This method takes account of errors occurring from omission of a word or incorrect ordering of word. Accuracy-addition and omission (%) was calculated as: *Number of correct words read/(14 + any additional words stated*) × *100*. This method, similar to the method used by Ramani, Police and Jacob (2014), allows other mistakes including, repetition or the addition of another word or sentence to reduce the accuracy score.

Reading speed was analysed using parametric statistical analysis in StatView, as the data was taken from a normally distributed sample. Reading accuracy results were analysed using non-parametric statistical analysis in StatView, as the data was not normally distributed. This was due to an evident ceiling effect for the reading accuracy scores, as a high proportion of the participants achieved the maximum score.

## Results

Twenty-four participants were included, mean age of 20.1 (SD 2.7) years.

### Reading speed

**Figure 1:**
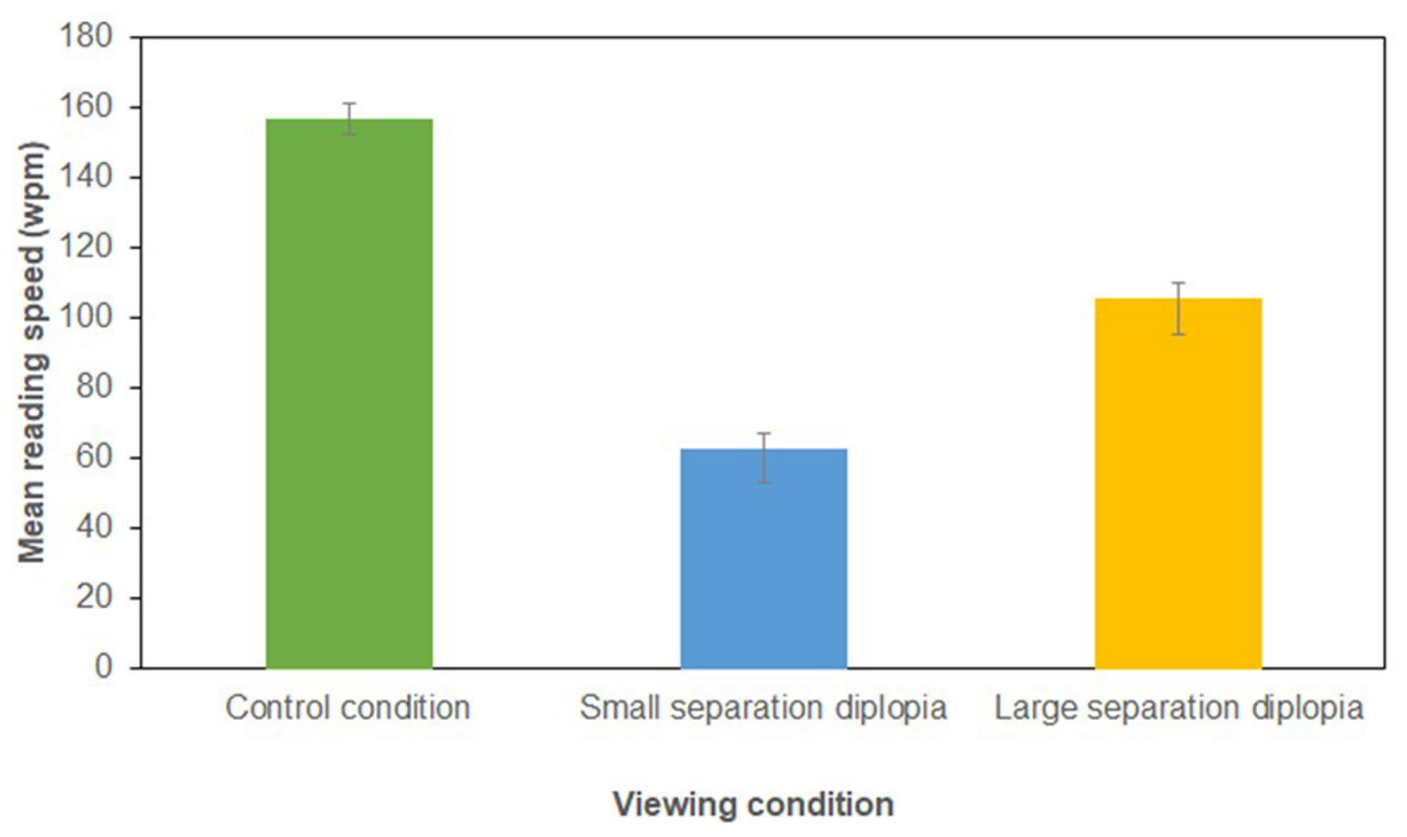
The mean and standard error reading speed (words per minute) for each viewing condition.

The mean reading speed in the control condition was (156.90 wpm, standard error (SE): 4.51). Compared to the control condition reading speed was reduced in both the small separation diplopia condition (62.75 wpm, SE: 9.53) and large separation diplopia condition (105.71 wpm, SE: 0.00) (Figure 1).

A one factor repeated measure ANOVA showed that viewing condition had a statistically significant impact on mean reading speed (F_2,46_ = 37.770, p < 0.0001). Reading with small separation diplopia caused a 60.01% (94.15 wpm) reduction in mean reading speed compared to the control condition, this difference was statistically significant (t(23) = 9.873, p < 0.001). Reading with large separation diplopia caused a 32.63% (51.19 wpm) reduction in mean reading speed compared to the control condition, this difference was statistically significant (t(23) = 4.897, p < 0.001). Reading with small separation diplopia reduced the mean reading speed by 40.64% (42.96 wpm) compared to the large separation diplopia condition, this difference was statistically significant (t(23) = 3.477, p < 0.01).

### Accuracy-omission

**Figure 2:**
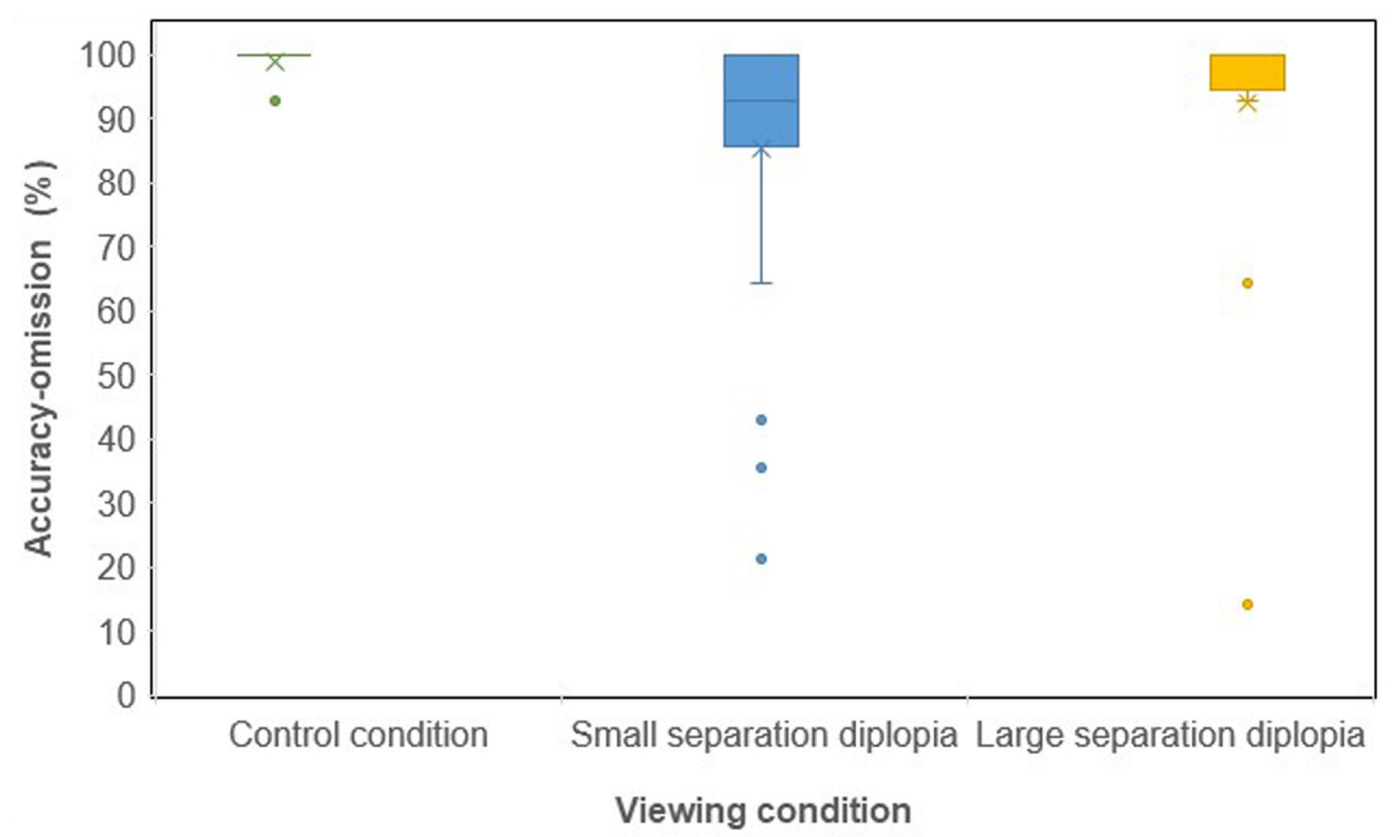
The median and interquartile ranges of the accuracy-omission (%) for each viewing condition.

The median and interquartile range (IQR) of the accuracy-omission (%) for each viewing condition are shown in Table 1 and Figure 2.

**Table 1:** Percentage accuracy-omission results for the control, small separation diplopia and large separation diplopia conditions.

	Accuracy-omission (%)		

	Control	Small separation diplopia	Large separation diplopia

Median	100	92.86	100
Q1	100	85.71	98.21
Q3	100	100	100
IQR	0	14.29	1.79
Mean	99.11	85.42	92.56

A Friedman test showed the effect of viewing condition on the median accuracy-omission (%) was statistically significant (p < 0.01). Reading with large separation diplopia and with the control condition resulted in a median accuracy-omission of 100%, however the large separation diplopia condition results were more variable (larger IQR). The difference between the accuracy-omission (%) results for the large separation and control conditions was statistically significant (p < 0.05) (Wilcoxon Signed Rank Test). Reading with small separation diplopia significantly reduced the median accuracy-omission by 7.14% compared to the control condition (p < 0.01) and by 7.14% compared to the large separation diplopia condition (p < 0.05) (Wilcoxon Signed Rank Test).

### Accuracy-addition and omission

**Figure 3:**
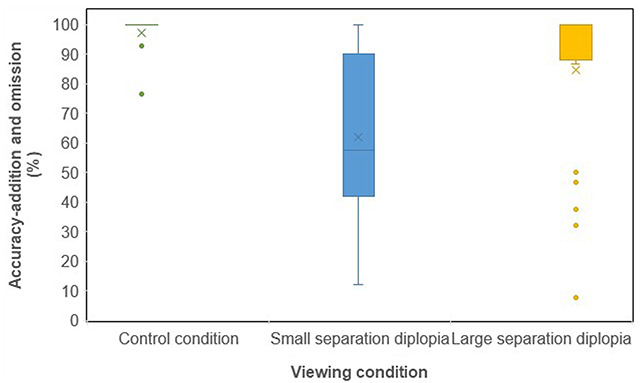
The median and interquartile ranges of the accuracy-addition and omission (%) for each viewing condition.

The median and IQR of the accuracy-addition and omission (%) for each viewing condition are shown in Table 2 and Figure 3.

**Table 2:** Percentage accuracy-addition and omission results for the control, small separation diplopia and large separation diplopia conditions.

	Accuracy-addition and omission (%)		

	Control	Small separation diplopia	Large separation diplopia

Median	100	57.50	100
Q1	100	43.16	91.31
Q3	100	84.98	100
IQR	0	41.82	8.69
Mean	97.22	62.12	84.71

The Friedman test showed that viewing condition had a statistically significant effect on accuracy-addition and omission (%) (p < 0.0001). Reading with large separation diplopia and with the control condition resulted in a median accuracy-addition and omission of 100%, however the large separation diplopia condition results were more variable (larger IQR). The difference between the accuracy-addition and omission (%) results for the large separation and control conditions was statistically significant (p < 0.05) (Wilcoxon Signed Rank Test). Reading with small separation diplopia significantly reduced the median accuracy-addition and omission by 42.50% compared to the control condition (p < 0.001) and by 42.50% compared to the large separation diplopia condition (p < 0.05) (Wilcoxon Signed Rank Test).

## Discussion

Inducing vertical diplopia in participants with BSV using Fresnel prisms reduced reading speed and reduced reading accuracy compared to the control condition. The finding that induced vertical diplopia with Fresnel prisms reduces reading speed and accuracy supports the self-reported impact of diplopia on reading found in previous studies (Hatt et al. 2009a; Hatt et al. 2009b; Wang et al. 2013). As reading is an integral part of study and employment, the findings also support the self-reported negative impact of diplopia on work or study found by Lin, He and Xiao (2015).

The results of this study suggest that both the presence and separation of diplopia should be taken into account when considering the functional impact of diplopia. Induced vertical diplopia of smaller separation caused a greater reduction in reading speed and accuracy than the larger separation diplopia. Mean reading speed in the control condition was 156.90 wpm whereas in the large separation it was reduced to 105.71 wpm, a 51.19 wpm or 32.63% reduction. Compared to the control condition, reading speed in the small separation diplopia condition was reduced to 62.75 wpm, a 94.15 wpm or 60.01% reduction. The differences between each of these reading speeds were statistically significant. Whittaker and Lovie-Kitchen (1993) reported that a reading rate of 80 wpm was needed for sustained reading, which suggests sustained reading may be possible in the presence of large separation diplopia, but not small separation diplopia.

Reading speed has been shown to be affected more than accuracy in some studies of reading (Ramani, Police & Jacob 2014), whilst some consider both measures to be important when evaluating reading (Beasley & Davies 2013) and others have reported a correlation between improved reading speed and improved reading accuracy (Jeanes et al. 1997). There is no accepted single method of measuring reading accuracy and different methods have been reported (Jeanes et al. 1997; Birch et al. 2010; Ramani, Police & Jacob 2014; Vurro, Crowell & Pezaris 2014). Reading accuracy was calculated using two different methods in this study, ‘accuracy-omission’ and ‘accuracy-addition and omission,’ as the authors believed the diplopia conditions may create word addition errors. Both methods of measuring reading accuracy showed a statistically significant reduction in reading accuracy (%) for the small separation diplopia condition compared to the control condition, 7.14% accuracy-omission (p < 0.01) and 42.50% accuracy-addition and omission (p < 0.001). Both methods of measuring reading accuracy also showed a statistically significant reduction in reading accuracy (%) for the small separation diplopia condition compared to the large separation diplopia condition, 7.14% accuracy-omission (p < 0.05) and 42.50% accuracy-addition and omission (p < 0.05). Despite the control condition and the large separation diplopia condition median accuracy results being 100%, the difference between the results was found to be statistically significant for both methods of measuring accuracy (p < 0.05), because the large separation diplopia condition results were more variable and had a larger IQR. Incorporating the addition of incorrect words, as well as the omission of words, into the measurement of reading accuracy seems justified, particularly when measuring the effect of diplopia on reading was the aim of the study. The greater reduction in reading accuracy using the accuracy-addition and omission method reflected the frequent repetition, or addition of incorrect words that occurred in the small separation diplopia condition.

Given that a larger separation of diplopia had a considerably smaller impact on reading speed and accuracy, the use of Fresnel prisms to further separate diplopia would be an appropriate conservative management option if BSV cannot be achieved. Five NHS trusts in England had online information leaflets explaining the use of prisms, however there is little evidence that prisms are currently used in this way. All five (Sherwood Forest NHS Trust 2012; Macintosh 2014; Holmes-Smith 2015; Maidstone and Tunbridge Wells NHS Trust 2015; North Devon Healthcare NHS Trust 2016) mentioned prisms could be used to join the diplopia, but only one (Maidstone and Tunbridge Wells NHS Trust 2015) stated that prisms could be used to further separate diplopic images. The results of this study suggest that such a use should be considered as a treatment option. Currently occlusion is the chosen method to temporarily relieve diplopia symptoms when BSV cannot be achieved, yet an interesting area for future study would be to measure the effect of monocular occlusion on reading speed and accuracy.

This study has several limitations. Given that vertical diplopia was induced with Fresnel prisms rather than occurring as a genuine symptom, the results may not be exactly replicated in a population of patients with diplopia symptoms or other types of diplopia. We also acknowledge that reading comprehension was not measured in this study; an important consideration in future studies (Birch et al. 2010). The participant perception of which diplopia symptoms were worse was also not considered, but would be an important consideration in future studies to allow the interpretation of worsening or improving diplopia to be investigated. Finally, it is acknowledged that a student population is not always a representative population sample and orthoptic students in particular may differ from naïve students, as they have more experience of orthoptic clinical tests (Horwood & Riddell 2010; Zaman et al. 2013). However, the audio recording of the task and the experimenter being blind to the visual conditions during data analysis allowed accurate timings and unbiased analysis of the errors made during reading.

## Conclusion

Induced vertical diplopia with Fresnel prisms caused reading to be significantly slower and less accurate than no diplopia, when measured with the RRC. Smaller separation vertical diplopia (6Δ total vertical Fresnel prisms) caused reading to be significantly slower and less accurate than larger separation vertical diplopia (12Δ total vertical Fresnel prisms). Using Fresnel prisms to further separate diplopia should be considered as a treatment option when diplopia cannot be joined.
